# Methadone Maintenance Therapy in Vietnam: An Overview and Scaling-Up Plan

**DOI:** 10.1155/2012/732484

**Published:** 2012-11-25

**Authors:** Tam T. M. Nguyen, Long T. Nguyen, Manh D. Pham, Hoang H. Vu, Kevin P. Mulvey

**Affiliations:** ^1^Vietnam Administration of HIV/AIDS Control (VAAC), Ministry of Health, Ha Noi, Vietnam; ^2^Substance Abuse and Mental Health Services Administration, Center for Substance Abuse Treatment, Rockville, MD 20857, USA

## Abstract

Vietnam is among the countries with the highest rate of HIV transmission through injecting drug users. HIV prevalence among injecting drug users is 20% and up to 50% in many provinces. An estimated number of drug users in the country by the end of 2011 were 171,000 in which the most common is heroin (85%). Detoxification at home, community, and in rehabilitation centers have been the main modalities for managing heroin addiction until Methadone Maintenance Treatment (MMT) was piloted in 2008. Recent reports have demonstrated positive treatment outcomes. Incidence of HIV was found remarkably low among patients on MMT. Treatment has significantly improved the quality of life as well as stability for society. The government has granted the Ministry of Health (MoH) to expand Methadone treatment to at least 30 provinces to provide treatment for more than 80,000 drug users by 2015. The Vietnam Administration for HIV/AIDS Control (VAAC) and MOH have outlined the role and responsibility of key departments at the central and local levels in implementing and maintaining MMT treatment. This paper will describe the achievements of the MMT pilot program and the scaling-up plan as well as strategies to ensure quality and sustainability and to overcome the challenges in the coming years.

## 1. Introduction

As the end of May 2012, Vietnam had 171,000 registered drug users, in which 85% is Heroine users [1]. Drug use prevention and treatment has been a top priority of the Party and Government. Both supply and demand reduction programs have yielded the encouraging results. However, the achievements are just the first step, and there are many shortcomings and problems ahead. The relapse rate and number of drug-related crimes remain high.

Vietnam is among the countries with the highest rate of HIV transmission through drug injection [2]. The prevalence of HIV among injecting-drug users is 20% and cases attributable to drug injection account for 60%. The current rate of HIV in IDUs has reduced but remained high in some localities, namely, Dien Bien (56%), Quang Ninh (56%), Hai Phong (48%) and Ho Chi Minh (46%), Dong Nai and Nghe An (24%), Hanoi (21%), and Lao Cai (22%) [3].

Methadone has been proved as an effective medication for opioid dependence [4]. Methadone together with psychosocial therapies and support of the family and community has helped improve the health of drug users, reduce the crimes and rate of HIV transmission and other blood-borne diseases [5].

The guidance for implementation of the Law on HIV/AIDS stipulates Methadone is one of the measures to prevent HIV transmission among injecting drug users [6–8]. Per Ministry of Health's guideline on Opioid Dependence Treatment, Methadone serves three main purposes [9]: reduce the harms caused by using opioids, including HIV, hepatitis B, hepatitis C resulting from needle sharing, death from overdose and related criminal activities; reduce the illicit drug use and injection; and improve the quality of life of the addict. Per operation guidance, only registered addicts who are selected by the provincial selection committee are entitled for MMT treatment.

Hai Phong and Ho Chi Minh have been the two cities selected for the MMT pilot since 2008 [10]. Based on the results of the pilot project, the Government has allowed the expansion of the program to other cities. Hanoi was the third city in the country to provide MMT treatment for patients since the end of 2009. As of 30 June 2012, the national program has provided treatment for nearly 10,000 patients in 12 cities and provinces.

## 2. Data and Methods

We use available reports and documents to describe the implementation process and results of the program over the last four years. We refer to current policies which have important impacts on the MMT treatment program. We summarize the scaling-up plan and solutions for the anticipated challenges. We also refer to articles, reports, and guidance archived on MMT programs in other settings for discussion.

## 3. Results and the Scaling-Up Plan

After three years of implementation, Methadone treatment program in Vietnam has received strong supports from the Party, Government, and Ministries at all levels.  Table 1 shows the number of patients and treatment clinics of 11 among 63 provinces and cities up to 30 June 2012. The most mature clinics started induction for patients in April 2008. The total of 44 MMT clinics has been providing Methadone treatment for 9,611 patients. Besides taking Methadone every day, patients benefit from variety of auxiliary services including counseling, accessing clean syringes and needles, condom, peer education, group and family meetings, HIV counseling testing, referral to Antiretro Viral Treatment (ARV), and other treatments when needed. 

Independent studies and evaluations have showed positive achievements and have been a foundation to expansion of the pilot program. From the beginning, an ongoing cohort of 965 patients in Hai Phong and Ho Chi Minh City (HCMC) has been followed up and data available have been analyzed at third, sixth, and ninth month. The median age for the study population was 30 years (range from 16.6 to 58), the majority were males (95%). Most patients were living with their parents or siblings at the time of treatment initiation (84%), 37% living with spouse and children, and less than 10% staying with relatives or friends. Most patients were at low educational level, with less than 56% completed secondary education. Just over 27% were able to perform basic tasks and only 11% were employed at the time of the study, usually by the family business. Twenty-one percent (205) of patients were HIV, positive, and 16% (33) of them were on ARV by the ninth month on MMT.

After nine months, retention in the MMT treatment program was 90% [11]. The number dropped was mainly those were arrested due involvement in drug dealing and those dying of AIDS. The rate of patients failed to take Methadone once at least within 5 days or more is low (1%). The main causes of this adherence deficiency are the work condition and distance from home that do not favor the patients to visit clinic on a daily basis. Prescribed MMT dose varies among patients. The lowest and highest daily doses are 5 mg and 470 mg, respectively, with an average of 109 mg per day. Dose also differs between patients on ARV treatment (150 mg) and not on ARV (100 mg).

Risk behaviors among patients under treatment have been reduced significantly. After 9 months, among patients who continued to use drug, only 56% of injected compared with 87% did before treatment. Only one patient reported sharing needle while 11%–30% before treatment. Almost (96%) of patients reported consistently use of condoms when having sex with sex workers. One new HIV seroconverted case found among 760 negative patients after nine months. This rate is much lower than the estimated HIV incidence among drug users before having the MMT pilot [12]. Testing result also showed that 9 and 72 patients had hepatitis B and hepatitis C, respectively. However, the confirmation of the result could not exclude the impact of the window period.

Quality of life of the patient has improved significantly. A study using the Kessler Psychological Distress Scale (K10)'s scale [13] showed the improvement in both physical and mental health. Before treatment, 80% of patients had a score greater than 15, the level of anxiety or depressive disorders and this rate dropped to 10% after nine months of treatment [14]. According to reports by the local police, the program has showed remarkably impact on the community's security where the clinic operated [15]. Meanwhile, the program has involved the local police to ensure patients adhere to the regulations set by the treatment program. It has been seen that conflicts within family and neighborhood have reduced to below 1% compared with 41% reported before addicts commenced the treatment [14]. Treatment has helped patients and their families save a remarkable amount of money previously used for buying drug, detoxification, and healthcare. Tran B showed cost-effectiveness of the treatment program in both medical and social aspects in the context of HIV/AIDS prevention, care, and treatment programs [16, 17]. At the same time, a rapid evaluation in Hai Phong has demonstrated that the investment in Methadone program significantly reduced costs compared to the compulsory detoxification model [18].

Monitoring and evaluation of technical activities have been carried out routinely. From the start of the pilot, MoH approved a protocol to evaluate the treatment effectiveness. Results have been publicized and shared periodically and widely among staff, patients, and policy makers. At the same time it has collaborated with different agencies and organizations to step by step finalize tools based upon national and international treatment standards. At the provincial level, upon having a consensus for implementation of MMT program, the People Council and People Committee has granted Department of Health, Labor and Public Security authorities and responsibilities to implement specific activities at all levels. The Department of Health has been the contact point to provide direction, coordination, and implementation of the program.

All activities have been closely coordinated by MoH through a national MMT technical working group. The group comprised of leaders and officers from VAAC, medical and social academic institutions, mental health hospitals, local and international donors, and organizations. Group members brought the technical support needs for VAAC to build and coordinate monthly technical support plans to each clinic. The United States President Emergency for AIDS Relief or PEPFAR has been the major donor for HIV/AIDS prevention, care, and treatment, including for the Methadone program. The United Kingdom's Department for International Department (DFID), World Bank, and Global Fund have supported facilities, staff and running costs and recently medication. The central and local governments have planned for an increase in the level of investment for HIV/AIDS services, including MMT to supplement the decline in the international funding. It has been clear that MOH and the international donors have been working in a principle of one national strategy and transparency to increase the impact of resources.

After reviewing the results of the pilot program, the government approved the expansion plan proposed by the MOH. Hanoi was the third city in the country has been approved to provide MMT treatment since the end of 2009. By then, nine provinces have proposed and had permission to implement the program. The target of 67 MMT clinics is set to provide treatment for 15,600 patients in 13 provinces by the end of 2012. MOH expected around 80,000 patients will be treated in 245 clinics by 2015. Total expenses estimated for the 2011-2012 period is USD 8.5 million. Methadone production domestically has been considered to use for the 2010–2015 period. It is estimated that the total amount of Methadone needed for this period is 1,724,990 liters with a concentration of 10 mg per mL, including 118,078 liters for 2010–2012 period.

MoH has directed relevant departments and worked with various ministries in the development of the Decree on Methadone maintenance treatment. This creates needed framework and terms for increasing access, expanding program, ensuring the sustainability and quality. It also helps increase the autonomy of provinces in implementing the program while following the standards and regulations set by the central government. Ultimately, the provincial departments of health will regularly inspect and supervise the implementation. Expenses for building or renovating clinics as well as hiring staff will be borne by provinces.

While efforts have been spent for ensuring quality of treatment, significant movement in policies has been recognized regarding the promotion of community-based treatment. For the last five years, the government has expressed a desire to develop effective and friendly drug management modalities [6]. Accordingly, it proscribed opening of new compulsory detoxification centers and at the same time requested Ministry of Labor Invalids and Social Affairs (MOLISA) to develop a renovation project on drug treatment [19]. The ministry saw a number of opportunities for Vietnam in evolving community-based treatment services for drug users as well as HIV/AIDS. The first fee to pay MMT model managed by the Hai Phong Department of Labor Invalids and Social Affairs was approved to operate in 2011. Patients have received services similar to the clinics run by the Ministry of Health and agreed to pay less than half a dollar for the clinic to cover the operating expenses.

The recently approved plan to develop social work [20] has been the paramount foundation for having continuum of services for drug treatment and HIV/AIDS. Budgeted for over one hundred million dollars using almost central and local resources, MOLISA is assigned to lead this plan. Among restructuring human resources and building capacity for social work professionals, at least ten community-based centers will be established. HIV and drug treatment among other services will be provided in these centers.

## 4. Discussion

Medicated Assisted Therapy using Methadone has been applied around the world for many decades. Mattick et al. summarized epidemiologic research that shows the effectiveness of Methadone treatment. For Vietnam, it is not an exception that the program has brought benefits for patients and communities and has helped prevent HIV transmission. Methadone helps reduce the long term effects of opioid use and assists patient gradually return to a normal life. The program has integrated ancillary services that are critical in achieving the treatment outcomes [21]. Sufficient dose could have been associated with better retention rate [22]. The drastic direction and guidance from the Party and Government combined with the technical and financial assistances from donors are central to the successes of the program. 

There are many challenges ahead in the expansion and maintenance of the program. The need for treatment is huge but the program capacity is limited. This creates an enormous pressure on the treatment facility and staff as well as the healthcare system. The proportion of patients treated by the end of 2012 is only 9% those need treatment, a low coverage index set by WHO [23]. The professional knowledge and skills on addiction treatment are limited. Most of the staff has no prior experience in general medicine and addiction in order to examine and communicate with patients comprehensively. Addiction treatment, particularly MMT is still the new field in Vietnam so the assistance for capacity building is always critical. While in-service training has been well implemented, integrating preservice training into course work for medical and social professionals could help sustain capacity for the field.

Although the two epidemics are interrelated, the policies on drug and HIV/AIDS prevention and treatment are not yet harmonized to maximize the advantages of services. The current addiction management modalities with limited evidence [24, 25] are the barriers to the treatment access. The role of local police is important in the overall drug control program; however, the decision on who should be on treatment should be determined using the standardized diagnosis. More information on the nature of addiction and recovery is needed to encourage access to treatment services. Yet the negative perceptions of community on drug users and regulations on drug rehabilitation make patients hesitant and fear to participate in any community-based treatment services, including MMT. They are often at risk of being sent to compulsory treatment if considered being bad users [26].

Nevertheless, significant progress has been made to facilitate the expansion. The recently released Law on Handling of Administrative Sanctions is the important legal framework to promote drug treatment in the community. In addition, treatment planning will include medical and drug dependence professionals in filing the case. Therefore, preparation for treatment services in the community, including MMT is urgently needed. Second, the government has aimed to strengthen the National Committee on Drug, HIV, and Prostitution from the central to local governments. This is an important strategy to promote collaboration and coordination of programs. Significant central and local budget has been approved for national target programs for the period 2012–2015. The allocation of budget shows the commitment of the government toward sustaining the achievements and ensuring the quality of services. Finally, there is a consensus from the national committee, ministries, provinces, and donors in harmonizing the resources. This is important in maximizing the use of resources in order to expand the available evidence-based practices.

## 5. Conclusion

For the past five years, the Government of Vietnam has given direction to maintain, sustain, and expand the MMT program after the successful pilot. MMT is among the key components of the HIV/AIDS program that the government has committed to allocate resources. Special attention has been paid to human and financial resources in the context of the decline in external funding. MOH, relevant ministries, institutions, and international partners have been working well together to plan, implement, and monitor MMT program at all levels. The national renovation plan on the compulsory rehabilitation modality is an important driving motivation for drug treatment. The current barriers to the treatment access will be resolved as a result of the expansion of the MMT program. The systematic integration of the Methadone treatment into primary health care and existing services, such as HIV/AIDS and mental health will be cost efficient. The consensus among all is the foundation to ensure the sustainability of the program. Close collaboration and coordination in the implementation of the MMT program will continue to yield safe and secured services for patient, clinic staff, and the community. These will help maximize the use of resources for scaling-up and ensuring quality of treatment.

## Figures and Tables

**Table 1:** Number of patients on MMT from August 2008 to June 2012.

Number	Province	Date operated	Clinic name	Number of patients on treatment
1	Hai Phong	4/2008	Le Chan	545
		4/2008	Thuy Nguyen	401
		8/2008	Ngo Quyen	293
		4/2011	Hai an	224
		6/2011	An Duong	317
		6/2011	An Lao	221
		7/2011	Hong Bang	196
		7/2011	Kien An	286
		3/2012	Duong Kinh	50
		6/2012	An Hung	20

			Subtotal	2553

2	Ho Chi Minh	5/2008	District 4	276
		5/2008	District 6	284
		5/2008	Binh Thanh	278
		1/2011	Thu Duc	141
		3/2011	District 8	278

			Subtotal	1257

3	Hanoi	12/2009	Tu Liem	247
		7/2010	Long Bien	239
		2/2011	Son Tay	109
		10/2010	Ha Dong	210
		11/2011	Hai Ba Trung	182
		5/2012	Dong Da	42

			Subtotal	1029

4	Can Tho	6/2010	Cai Rang	242
		6/2010	Ninh Kieu	240
		11/2011	O Mon	92

			Subtotal	574

5	Da Nang	9/2010	Clinic 1	129
		7/2011	Clinic 2	93

			Subtotal	222

6	Hai Duong	11/2010	Hai Duong City	268
		11/2010	Chi Linh	161
		10/2011	Kim Thanh	91
		7/2011	Kinh Mon	191

			Subtotal	711

7	Dien Bien	3/2011	Thanh Xuong	295
		10/2011	Noong Bua	240
		10/2011	Muong Ang	154
		3/2011	Tuan Giao	215

			Subtotal	904

8	Nam Dinh	3/2011	Nam Dinh	235
		7/2011	Giao Thuy	230
		7/2011	Xuan Truong	249

			Subtotal	714

9	Thanh hoa	5/2011	Thanh Hoa City	387

			Subtotal	387

10	Thai Nguyen	10/2011	Dai Tu	262
		10/2011	Dong Hy	263
		11/2011	Pho Yen	248
		10/2011	Trung Thanh	147
		11/2011	Tuc Duyen	146

			Subtotal	1066

11	Quang Ninh	11/2011	Cam Pha	194

			Subtotal	194

Total number of clinics = 44				9,611
